# Did private election administration funding advantage Democrats in 2020?

**DOI:** 10.1073/pnas.2317563121

**Published:** 2024-05-21

**Authors:** Apoorva Lal, Daniel M. Thompson

**Affiliations:** ^a^Independent Researcher, Redwood City, CA 94063; ^b^Department of Political Science, University of California Los Angeles, Los Angeles, CA 90095

**Keywords:** election administration, political economy, synthetic control

## Abstract

Private donors contributed more than $350 million to local election officials to support the administration of the 2020 election. Supporters argue these grants were neutral and necessary to maintain normal election operations during the pandemic, while critics worry these grants mostly went to Democratic strongholds and tilted election outcomes. How much did these grants shape the 2020 presidential election? To answer this question, we collect administrative data on private election administration grants and election outcomes. We then use advances in synthetic control methods to compare presidential election results and turnout in counties that received grants to counties with similar election results and turnout before 2020. While Democratic counties were more likely to apply for a grant, we find that the grants did not have a noticeable effect on the presidential election. Our estimates of the average effect on Democratic vote share range from 0.03 to 0.36 percentage points. Our estimates of the average effect of receiving a grant on turnout range from 0.03 to 0.14 percentage points. Across specifications, our 95% CIs typically include negative effects and all fail to include effects on Democratic vote share larger than 0.58 percentage points and effects on turnout larger than 0.40 percentage points. We characterize the magnitude of our effects by asking how large they are compared to the margin by which Biden won the 2020 election. In simple bench-marking exercises, we find that the effects of the grants were likely too small to have changed the outcome of the 2020 presidential election.

## Significance

Private donors contributed more than $350 million to local election officials to support the administration of the 2020 election. Supporters argue these grants were neutral and necessary to maintain normal election operations during the pandemic, while critics worry these grants mostly went to Democratic strongholds and tilted election outcomes. These concerns have led 24 states to restrict private election grants. We find that, while counties that favor Democrats were much more likely to apply for a grant, the grants did not have a noticeable effect on the 2020 presidential election.

Private donors contributed more than $350 million to local election officials to support the administration of the presidential election in 2020 (1, 2). This nearly matches the supplemental funding Congress appropriated to support local election administration in 2020 (3) and is a substantial share of the $2 to $3 billion spent in a typical national election (4, 5). The private donors and their supporters argue this money was necessary to ensure all eligible citizens had a chance to vote amid the disruptions caused by the COVID-19 pandemic (6). Many local election officials echo this view with one anonymous official saying they used the funding to “alleviate choke points and barriers to voting” (7). Favoring these arguments, Michigan voters approved an amendment to their state’s constitution in 2022 that protects the right of local governments to receive private funding for election administration.

Critics of these donations argue that private donors overwhelmingly favored Democratic-leaning counties and municipalities and that these grants have the potential to tilt elections in favor of one party by increasing the participation of some citizens more than others (8). In one complaint filed before the Federal Election Commission, the plaintiff points out that a large share of the funds donated in 2020 went to Democratic-leaning parts of the country and alleges that the donors have a “hidden motive to increase Joe Biden’s statewide vote” (9). These concerns led twenty-four states to adopt laws banning or limiting private donations to local election officials (10). How much did private election administration grants tilt the 2020 election?

We address this question by combining county-level administrative data on turnout, presidential voting, election spending, and election administration with records of which county governments received a private donation from the largest private election administration donor in 2020, the Center for Tech and Civic Life (CTCL). We document that counties that support Democrats were more likely to apply for private election funding in 2020, which is consistent with critics’ allegations. Since Democratic-leaning counties were much more likely to apply, and every eligible applicant received a grant, a simple comparison of turnout and presidential vote shares in counties that did or did not receive a grant fails to reveal the grant’s impact. We mitigate this bias by comparing grant-funded counties to those without funding but with similar pre-2020 turnout and voting trends using recent advancements in synthetic control methods (11).

We find that, despite the scale of the CTCL grant program in 2020 and the tendency of the money to go to Democratic-leaning counties, private funding did not noticeably advantage Joe Biden in the 2020 presidential election. We estimate that receiving a grant increased support for Democrats by between 0.03 and 0.36 percentage points and increased turnout by less than 0.14 percentage points. We validate our estimates using alternative machine learning and econometric approaches to estimating the effects, and these approaches produce similar estimates. We also replicate our analyses with a separate original dataset of municipal-level presidential election results in Wisconsin and the distribution of private grants in Wisconsin, finding that CTCL grants did not substantially increase turnout or Democratic vote share in Wisconsin either. Since large counties and counties in battleground states have a larger effect on the aggregate election outcome, we also estimate the effects in these counties separately. We find that grants had a similar effect in large counties and counties in battleground states. We also present evidence that the small average effects are not masking large effects for the small number of counties that received relatively large grants.

To characterize the magnitude of the effects, we compare our estimates to the state-by-state margins in the 2020 presidential election. We also conduct a simple analysis in which we remove the average effect. Despite the razor-thin margins in 2020, we find that the turnout and Democratic vote share effects are not large enough to have swung the election to Joe Biden in our simple simulations.

Beyond the ongoing policy debate about private election funding, this paper contributes to a growing social scientific literature on the effects of local election administration. Democratic and Republican officials often disagree over how much to spend on elections and how they should be funded (12, 13). This leads to a conventional wisdom that spending more or less will have substantial effects on the outcomes of elections. Yet, while some changes in state and local election administration can affect participation and alter the composition of the electorate, the turnout effects tend to be modest, and the compositional effects are often hard to predict (14–21). Further, despite large differences between the Democratic and Republican positions on how much money to spend on elections, Democratic local officials do not produce more turnout or higher Democratic vote shares than do Republican local officials (22). This paper advances this literature by evaluating a large increase in resources rather than a single policy change. If local election officials are motivated to increase participation^*^ and are well informed about what administrative changes will be most effective, providing them resources should have a larger effect on turnout than any individual policy change. Our findings suggest that there may be less room for increasing turnout with local administrative changes than previously expected.

This paper also contributes to the large literature on the role of money in American politics. A vast literature studies the effects of campaign finance and the influence of special interests (see, e.g., refs. 23 and 24). This project is especially closely related to a small but growing literature on the influence of business leaders in politics (25). Many reforms have sought to limit the influence of money in politics by changing who can spend how much money on which races (see, e.g., refs. 26, 27). While many public officials and members of the public are concerned that donations to support local election administration offer a new, previously untapped way to influence election results without giving directly to candidates, our results suggest this may not be a substantial risk.

When considering the implications of our results, it is important to note that our paper reports estimates of the average effect of additional election funds at the current margin. If, for example, election officials had much smaller budgets than in 2020, grants may help to maintain the most basic election functions and thereby have substantial effects on participation and election outcomes. More resources may also help local governments maintain better security measures or make voting more convenient. While we cannot measure this directly, many of the recipients said the grants helped them make their local election more secure.^†^

## 1. Private Election Administration Grants in 2020

### 1.1. CTCL’s 2020 Grant Program.

In fall 2020, Mark Zuckerberg and Priscilla Chan donated approximately $350 million to the CTCL, a Chicago-based nonprofit, to administer a grant program for local election administration.^‡^ The CTCL invited all local governments responsible for administering elections to apply for funding and gave the funding to every eligible government that applied.^§^ The grants were intended to offset election administration expenses incurred from June 2020 until December 2020 and supplement federal emergency funding from the CARES Act^¶^ that many advocates viewed as too little and too constrained.^#^ The CTCL determined the maximum amount of each grant based on the eligible voting population of each jurisdiction as well as other demographics. According to our calculations, CTCL gave the median grant-receiving county approximately $0.81 per voting-age resident. The typical local government spends approximately $8 per eligible citizen on elections (4), making this a roughly 10% increase in the typical recipient’s election funding in a normal year.

According to reports submitted to the CTCL, election officials intended to use the grants in a variety of ways to make election day run smoother, offer alternative ways to vote, and reduce COVID transmission (30). Officials reported using the money to hire poll workers and other temporary staff, purchase mail balloting equipment and supplies, obtain protective equipment such as masks, and purchase other standard election equipment. One election administrator told the CTCL that “this unprecedented voter participation simply would have crippled the administration of our elections with devastating effects if we were left with the limited available municipal funds” (7). While a complete accounting of how the grants were spent is not available, the Wisconsin Institute for Law & Liberty collected data on how the grant funds were used by local governments in Wisconsin. We present these data in *SI Appendix*, Fig. S2. We find that approximately 40% of the funds were spent on staff and 40% were spent on equipment such as voting machines, mail ballot processing devices, and ballot drop boxes.

### 1.2. Grant and Election Data.

We draw on administrative grant and election data to study the effect of private election administration grants in the 2020 election. We build our dataset of grant recipients using the CTCL’s 2020 tax filing which contains a list of every grant made under this program. We digitized this tax document and extracted all of the recipient names and grant amounts. We also compiled and cleaned county-level presidential election results from 1992 to 2020. These results were collected from secretaries of state and reported in Dave Leip’s Atlas of US Presidential Elections.^‖^

**Fig. 1.**
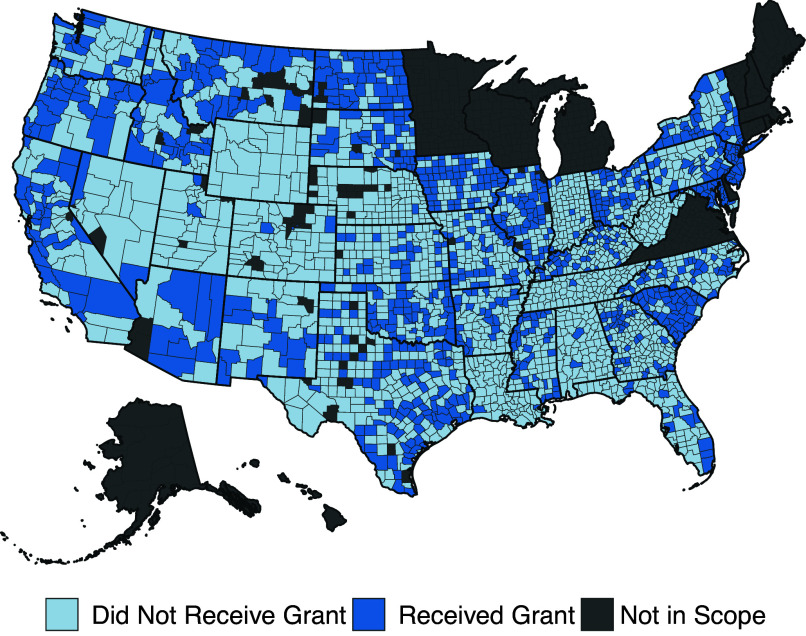
Geographic distribution of grants.

The official responsible for running elections varies across states and even, occasionally, within states (31). In ten mostly New England and Midwestern states, election administration is largely handled by municipal governments.^**^ In these states, we cannot distinguish between counties that did and did not receive grants because there are often many municipalities within a given county. To avoid incorrectly labeling these counties as receiving a grant when only a small portion of the county received a grant, we withhold these states from our analysis. We also withhold five counties with municipal election administrators in states where elections are typically run by county officials.^††^ Finally, we exclude 59 counties that either have fewer than 1,000 residents or have changing borders during our analysis period given the challenges associated with estimating turnout when the population estimate in the denominator of turnout will be noisy (Fig. 1).

### 1.3. Reasoning About the Effects of Election Administration Grants.

Should we expect grants like this to affect turnout and election results? One way to reason about this is to consider each of the changes in election administration that the grant money facilitates and evaluate who the change targets, how much it will increase participation in the targeted group, and how different the targeted and nontargeted groups are in terms of expected partisan voting. (This section draws heavily from ref. 32 and private conversations with the authors.)

Consider a grant-receiving county that spends the money on additional poll workers—more counties mentioned spending grant money on temporary staff or poll workers than on any other spending category (30). Staff may make the process of registering or requesting an absentee ballot easier, activities that are necessary for well-run elections but are unlikely to have substantial effects on participation. They may also help keep lines shorter. Suppose the additional staff reduce the average wait time by 10 min, a quite large effect. Pettigrew (33) finds that wait times decrease future turnout by approximately 1 percentage point for every hour a person waits. If this effect is linear and applies to people deciding whether to stay or leave based on line length, not just in future years, then reducing wait times by 10 min for the average voter increases participation by 0.17 percentage points. Imagine there are two polling places in a county with equal numbers of voters using each in a typical year. The local election official sends the new staff to only one location. Suppose further, as an extreme example, that 75% of voters in the precinct that received the extra staff typically vote for Democrats while only 25% of voters in the other precinct vote for Democrats. In this extreme example, overall turnout increases by 0.085 percentage points and Democratic vote share increases by an even smaller 0.021 percentage points. A substantial share of the funds was spent on activities that would most plausibly affect participation through wait times and in-person voting (e.g., hiring more poll workers, opening more polling places, etc.). Still, similar exercises using a similar approach for other common interventions—like adding ballot drop boxes (34), expanding mail voting (20)^‡‡^, adding polling places (15, 19, 36), and expanding early voting hours (17, 18)—all of which were also funded by CTCL funds, lead to the same conclusions. A large share of counties also reported spending money in ways that should have even smaller effects on participation and partisan balance like purchasing personal protective equipment for poll workers or purchasing new election equipment. Based on data from Wisconsin which we discuss in more detail in the appendix, approximately 68% of funds were spent in categories that might affect participation as described above (e.g., hiring staff, opening new polling places, creating a drive-thru voting option, buying equipment to scale up mail voting, and adding more ballot drop boxes), implying that the spending was not carefully tailored to have the greatest effect on participation.

Even after combining many small effects, this exercise leads us to expect that the effect of this grant money on turnout and Democratic vote share is quite small if there is any detectable effect at all.

## 2. Democratic-Leaning Counties Were More Likely to Apply for and Receive a Grant

One of the key concerns among critics of the private election administration grants is that Democratic-leaning counties were more likely to receive them. The CTCL gave a grant to every eligible county and municipality that applied.^§§^ If Democratic counties were more likely to apply and receive the money, and the money leads to higher turnout, the money can advantage Democrats when the state adds up the county totals.

**Fig. 2.**
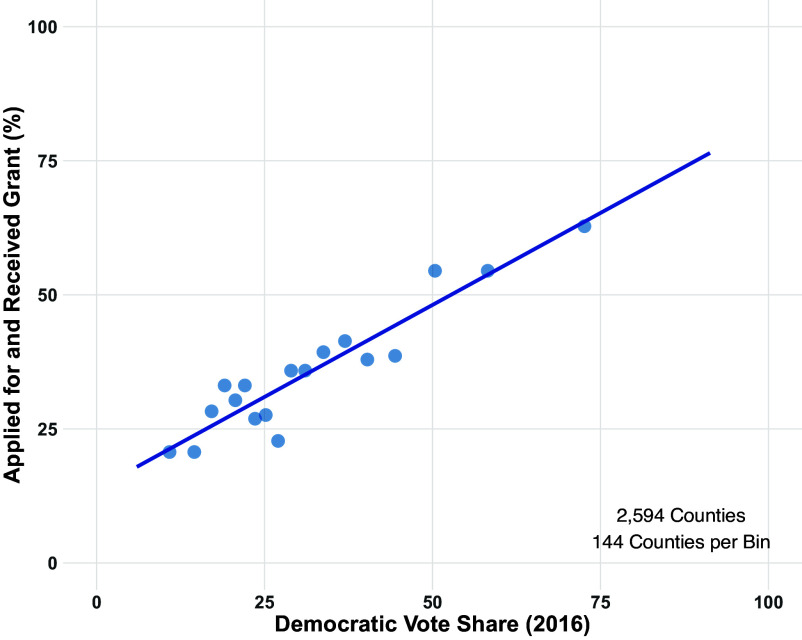
Democratic-leaning counties more likely to apply for and receive election assistance grants. Each dot represents the average of 144 counties binned based on two-party Democratic presidential vote share from 2016. The regression line is fit to the underlying county-level data.

Fig. 2 captures how much more likely Democratic-leaning counties were to apply for a grant than Republican-leaning counties. We use Democratic vote share in the 2016 presidential election as a measure of partisan lean. We find that the CTCL gave grants to about 30% of counties where Donald Trump received approximately 75% of the two-party presidential vote in 2016. Meanwhile, the CTCL gave to 65% of counties where Hilary Clinton received 75% of the two-party vote in 2016.

### 2.1. Why Did Democratic-Leaning Counties Apply at Higher Rates?.

Democratic-leaning counties differ from Republican-leaning counties in a large number of ways that could plausibly make them more likely to apply for a private election grant: They have larger populations, they are more densely populated, they are more racially and ethnically diverse, and they were more exposed to COVID-19 before the grants were announced on average. Might these characteristics account for the tendency of Democratic counties to apply for the private election grants at higher rates than Republican counties?

In Table 1, we document some simple descriptive patterns to help us understand how plausible are different explanations for the Democratic–Republican gap in private election grant applications. Each column presents coefficient estimates from a linear regression of grant receipt on 2016 Democratic presidential vote share and, in some cases, additional factors that might correlate with Democratic vote share and applying for a private election grant. We do not intend to estimate the causal effect of changing the share of Democrats in a county on the probability of applying for a grant. Instead, we use these regressions to evaluate plausible explanations for the Democratic–Republican gap in grant application rates.

**Table 1.** Unpacking the relationship between democratic vote share and grant application

	Applied for and received grant						
	(1)	(2)	(3)	(4)	(5)	(6)	(7)
Lag dem vote share	0.69 (0.06)	0.55 (0.07)	0.31 (0.07)	0.44 (0.10)	0.42 (0.10)	0.42 (0.10)	0.14 (0.15)
Log(Population)			0.06 (0.01)	0.06 (0.01)	0.06 (0.01)	0.06 (0.01)	0.05 (0.01)
Log(Median income)			0.10 (0.05)	0.06 (0.05)	0.06 (0.05)	0.06 (0.05)	0.04 (0.05)
Metro				0.03 (0.02)	0.03 (0.02)	0.03 (0.02)	0.06 (0.03)
Non-Hisp white share				0.16 (0.09)	0.13 (0.09)	0.13 (0.09)	0.13 (0.13)
COVID death rate					−0.00 (0.02)	−0.00 (0.02)	0.01 (0.04)
Social distancing share					−0.02 (0.03)	−0.02 (0.03)	0.00 (0.04)
NACo						0.01 (0.02)	0.01 (0.04)
Dem clerk							0.08 (0.03)
Constant	0.14 (0.02)					
Observations	2,594	2,594	2,593	2,592	2,537	2,537	1,068
State FEs	No	Yes	Yes	Yes	Yes	Yes	Yes

Robust standard reported in parentheses. Population is the voting-age population. Median Income is median household income measured with the 5-y ACS ending in 2019. Metro is an indicator for urban and suburban counties based on the Census nine-value urban–rural continuum. Non-Hisp White Share is the share of residents who are classified as non-Hispanic White in the 2020 census. COVID death rate is the number of deaths per 1,000 residents prior to September 1, 2020. Social Distancing Share is the share of Nationscape respondents in the county who report always complying with recommended social distancing in the early fall of 2020. NACo is an indicator for county membership in the National Association of Counties. Dem clerk is an indicator for a county having a Democratic local election official—limited to partisan officials.

As a point of reference, column 1 of Table 1 reports the coefficient on 2016 Democratic vote share from a simple bivariate regression. The coefficient implies that the probability of applying for and receiving a grant is 6.8 percentage points higher on average in a county with a 10-percentage-point higher Democratic vote share.

Some states were more favorable to private election grants while others actively discouraged counties from applying. For example, 23 states applied for private election grants from a similar grant program for state-level election officials (37). Some state officials also reached out to local officials encouraging them to apply while others threatened to sue local officials that applied.^¶¶^ In column 2 of Table 1, we include state fixed effects and find that the coefficient on 2016 Democratic vote share is approximately 19% lower after accounting for the tendency of Democratic counties to be in states where counties applied for the CTCL grant at a higher rate.

Among counties in the same state, Democratic counties tend to have more people, be more urban and racially diverse, be members of a national organization that informed counties about the grants, and have had worse experiences with COVID-19 in the spring and summer of 2020. We evaluate whether these differences between Democratic and Republican counties can account for the pattern of Democratic counties applying for the private election grants at higher rates. In column 3 of Table 1, we find that counties were 3.1 percentage points more likely to apply than other counties in the same state with the same populations and median incomes that voted 10 percentage points more for Republicans in 2016. In column 4, we add to the regression in column 3, adjusting for the non-Hispanic White share of the population and whether the county is urban or rural. After accounting for population, income, and the state a county is in, urban counties with a smaller share of non-Hispanic White residents vote more for Democrats and applied for the election administration grant at a lower rate. Accordingly, adjusting for the fact that Democratic counties are more urban and diverse, we find that the coefficient on 2016 Democratic vote share increases to 0.44. In columns 5 and 6 we make further adjustments to account for COVID-19 deaths and social distancing preferences prior to the 2020 election as well as membership in the National Association of Counties (NACo) which notified members of the grants. Accounting for these factors does not substantially change the coefficient on 2016 Democratic vote share.

In column 7, we introduce an additional measure of tendency to vote for Democrats–the fact that the county elected a Democrat to run elections. We find that counties with a Democratic local election official were more likely to apply for a CTCL grant compared to counties with a similar partisan lean, population, median income, urbanicity, racial and ethnic makeup, experience with COVID, and likely awareness of the grant program. When accounting for the party of the local election official, the relationship between past presidential vote share and grant receipt is considerably smaller. We also find that when this measure is included, the relationship between lagged Democratic presidential vote share and grant receipt is substantially smaller. This is unsurprising since places that support Democrats for president tend to elect Democrats as local government officials. This is consistent with two plausible explanations for why Democratic-leaning counties applied for CTCL grants at higher rates: Democratic local election officials may have anticipated higher costs in the 2020 election due to high demand for mail voting and other COVID mitigation measures, and Democratic local election officials were more motivated to apply or more likely to hear about it. We cannot distinguish between these explanations using these descriptive regressions.

Table 1 also suggests that counties with more capacity may have been more likely to apply for a CTCL grant. After accounting for many county-level factors, counties with larger and denser populations were more likely to apply for a CTCL grant. Large, dense counties tend to have more staff and be more professionalized^##^. While only speculative, this suggests counties with fewer staff and less available time to apply may have been less likely to apply.

Were counties more likely to apply for a CTCL grant when they anticipated higher costs due to COVID? In *SI Appendix*, section S3 in the online appendix, we present an additional analysis in which we compare the relationship between 2016 Democratic vote share and applying for a grant in states that dramatically expanded mail balloting in 2020 compared to states that severely restricted mail balloting or already mailed every registrant a ballot. This is potentially important because some states made expensive, rapid changes in how they ran elections, and local election officials in Democratic-leaning counties may have anticipated higher costs and been more motivated to apply for funding. [Many counties received federal emergency support for election administration through the CARES Act (29), but this funding was not, to our knowledge, greater in counties that made more election administration changes in 2020. So, even though the CARES Act may have reduced the absolute need for additional funds, it is unlikely that it changed the difference in the need between places implementing more versus fewer administrative changes.] The estimates are noisy and sensitive to which covariates we include in the regression, but the point estimates are consistent with some election officials in Democratic counties anticipating an increase in their costs associated with a transition to mail balloting, and applying for a grant to cover those costs.

## 3. Grants Did Not Substantially Increase Turnout or Democratic Vote Share

In this section, we detail our finding that the private election administration grants did not substantially increase turnout or Democratic vote share, and we explain how we estimate the effects of the grants. This section has five parts: First, we describe our estimation strategy and why it is appropriate for this setting. Second, we present graphical evidence that grants did not substantially increase turnout or Democratic vote share. Third, we report estimates of the effect of grants on turnout and Democratic vote share. Fourth, we document our independent analysis of grants to municipalities in Wisconsin which produces results similar to our main estimates. Fifth, we discuss alternative estimation strategies and document how all of these strategies yield similar results. Sixth, we present evidence that the effects are similar in more and less competitive states and more and less populous counties. Finally, we document that the effects are less positive in counties that received larger grants, suggesting that funding is not substantially affecting turnout or the composition of the electorate at the current margin.

### 3.1. Estimating the Effect of Private Grants in the 2020 Election.

**Fig. 3.**
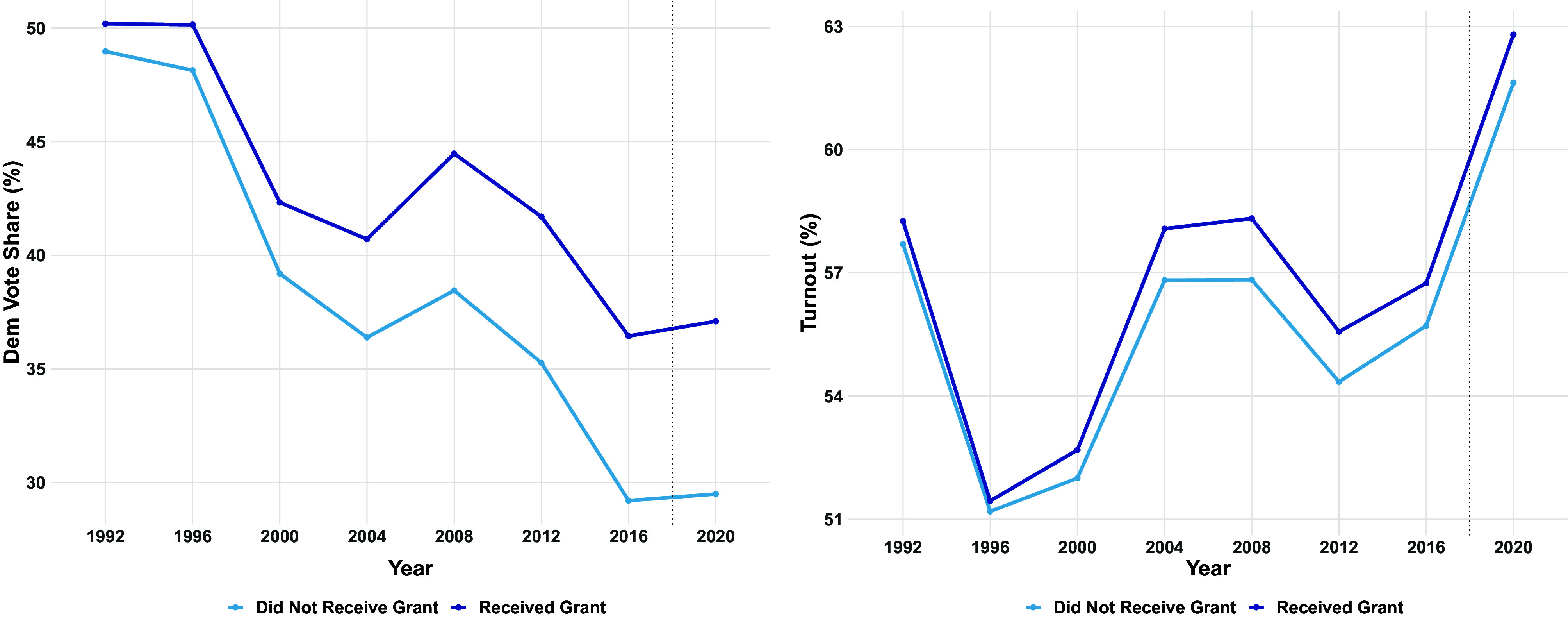
Democratic vote share was declining slower and turnout was higher in grant-receiving counties long before 2020.

Our goal in this section is to estimate the average effect CTCL grants had on turnout and Democratic vote share. As we document in Fig. 2, grant-receiving counties favored Democrats in 2016, 4 y before the grants were made. Given the tendency of counties to continue voting for the same party from one election to the next, we would expect grant-receiving counties to favor Democrats in the 2020 election more than counties that did not receive grants even if the grants had no effect on Democratic vote share or turnout. This type of selection can be accounted for using a difference-in-differences strategy, where we compare the difference between 2020 and pre-2020 turnout and vote share for Democrats in treated counties with the analogous difference in untreated counties. This approach would yield valid causal estimates under the assumption that turnout and Democratic vote share would have increased by the same amount in 2020 in treatment and control counties in the absence of the grants. Since we have measures of county-level turnout and Democratic vote share for many elections prior to 2020, we evaluate the plausibility of this assumption in Fig. 3. We find that Democratic vote share is decreasing slower in treated counties than in control counties. We also find that turnout is increasing faster in treated counties. These differences in treated and control trajectories suggest that simple difference-in-differences estimates will dramatically overstate the effect of grants on Democratic vote share and turnout.

To address the shortcomings with the standard difference-in-differences design in our setting, we follow ref. 11 in reweighting our difference-in-differences regressions with weights that ensure the treatment and control units are on similar trajectories prior to the treatment and make the pretreatment period as similar as possible to the posttreatment.^***^ This approach has three steps: 1) compute county weights that make the trend in the control units approximately equal to the trend in the treated units, 2) compute election weights that make the pretreatment period as similar as possible to the posttreatment period among control units, and 3) estimate a reweighted difference-in-differences regression weighting by the product of the county and election weights.

Formally, we compute county weights $\omega_{i}$ that minimize the difference between the outcome in the average treated county and a weighted average of untreated counties in elections prior to 2020

[1] $$\begin{array}{cc} \;{\text{argmin}}_{\omega_{0}\in R_{+},\omega \in \Omega } & \sum_{t=1}^{T_{\text{pre}}}{\left(\frac{1}{N_{\text{tr}}}\sum_{i=N_{\text{co}}+1}^{N}Y_{\mathrm{it}}-\omega_{0}-\sum_{i=1}^{N_{\text{co}}}\omega_{i}Y_{\mathrm{it}}\right)}^{2}\\ \; & +\zeta^{2}T_{\text{pre}}{\left||\omega |\right|}_{2}^{2}, \end{array}$$

where $Y_{\mathrm{it}}$ is the outcome in county i and election t, $T_{\text{pre}}$ is the number of pretreatment periods, N is the number of control and treated units, $N_{\text{co}}$ is the number of control units, Ω is the set containing all valid ω in which all $\omega_{i}$ fall between 0 and 1 inclusive and ω sums to one, and ζ is a regularization parameter proposed in ref. 11. Since the intercept $\omega_{0}$ is not regularized, $\omega_{0}$ represents the average pretreatment difference between the treated and control units and means that the county weights produce a weighted control mean that follows the same trajectory as the treatment mean but may not be at the same level (permitting this to be nonzero allows the synthetic difference in differences method to optimally interpolate between conventional difference in differences (nonzero $\omega_{0}$ with uniform $\omega =1/N_{\text{co}}$ weights) and synthetic control (data-driven ω, and $\omega_{0}=0$) based on the data).

We then compute time weights $\lambda_{t}$ that minimize a nearly identical expression:

$$\;{\text{argmin}}_{\lambda_{0}\in R,\lambda \in \Lambda }\sum_{i=1}^{N_{\text{co}}}{\left(\frac{1}{T_{\text{post}}}\sum_{t=T_{\text{pre}}+1}^{T}Y_{\mathrm{it}}-\lambda_{0}-\sum_{t=1}^{T_{\text{pre}}}\lambda_{t}Y_{\mathrm{it}}\right)}^{2}.$$

We use the product of these weights as the weights in a weighted difference-in-differences least squares regression

$$\;{\text{argmin}}_{\tau ,\alpha ,\beta }\sum_{i=1}^{N}\sum_{t=1}^{T}{\left(Y_{\mathrm{it}}-\alpha_{i}-\beta_{t}-W_{\mathrm{it}}\tau \right)}^{2}\hat{\omega_{i}}{\hat{\lambda }}_{t},$$

where $W_{\mathrm{it}}$ is an indicator for the treatment, $\alpha_{i}$ is a county fixed effect, $\beta_{t}$ is an election fixed effect, and τ is the treatment effect of interest. As with the classical synthetic control method (39) and generalized synthetic control method (41), the weighted regression coefficient $\hat{\tau }$ from the synthetic difference in differences method yields consistent estimates for the average treatment effect on the treated (ATT) under a low-rank approximation for the untreated potential outcome $Y^{0}$, which requires that election outcomes in the absence of the grants can be approximated by county intercepts, election year shocks, and low-rank time varying slopes at the county level. This design assumption is strictly weaker than the parallel trends assumption required by difference in difference methods, which we see is implausible from Fig. 3.

To validate the weighted difference-in-differences estimator, we study the 2016 presidential election as a placebo case. We do this by deleting 2020 from our data and pretending that the grants were handed out in 2016. We then rerun the weighted difference-in-difference procedure to produce estimates of the placebo effect. Since the grants were not handed out until 2020, an unbiased estimator will find placebo effects that are close to zero. We present these estimates in *SI Appendix*, section S5 in the online appendix. Consistent with the goal of the estimator, we confirm that the weighted difference-in-differences approach fails to estimate a statistically significant placebo effect.

### 3.2. Graphical Evidence that Grants Had Minimal Effect on Democratic Vote Share and Turnout.

**Fig. 4.**
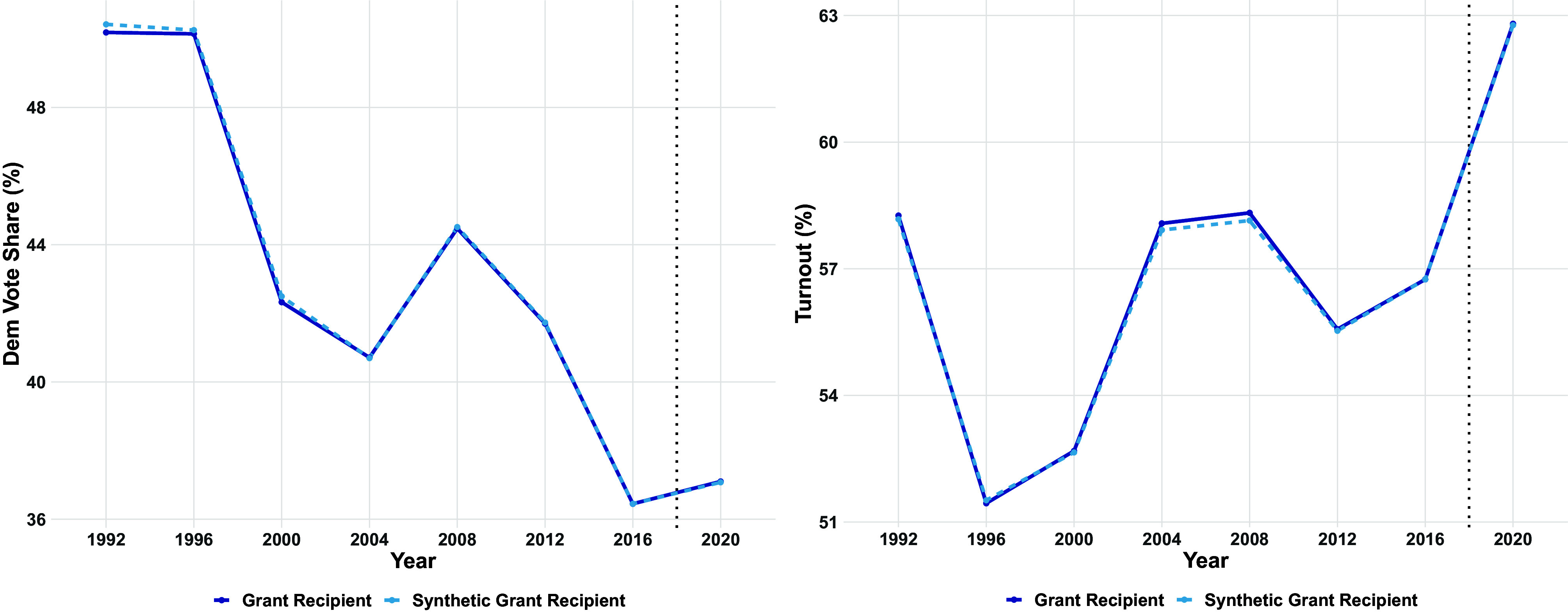
Trends in democratic vote share and turnout in grant-receiving and synthetic difference-in-differences counterfactual over time.

First, we present graphical evidence that CTCL grants did not substantially increase turnout or vote share for Democrats. Fig. 4 compares Democratic vote share and turnout for the average grant recipient over time to the counterfactual implied by synthetic difference-in-differences. Across both the Democratic vote share panel on the left and the turnout panel on the right, we see the synthetic difference-in-differences procedure produces a counterfactual that almost perfectly matches the average trajectory for grant recipients. The exceptions to this perfect match are in 1992 where counterfactual Democratic vote share is slightly higher than observed Democratic vote share and 2004 and 2008 where counterfactual turnout is slightly lower than observed turnout. In all three cases, the differences are small with gaps of less than 0.25 percentage points.

Fig. 4 also makes clear that any average effect of the grants is so small as to not be visible in the Democratic vote share or turnout plots. If there were a visible effect in either plot, it would appear as a difference between the grant recipient and counterfactual lines in 2020. Instead, these lines continue to almost perfectly overlap in 2020 just like they did prior to 2020, implying that the average grant did not substantially advantage either party or noticeably increase turnout.

In *SI Appendix*, Fig. S5 in the appendix, we build on these plots, showing the gap in Democratic vote share and turnout between the average grant-receiving county and its synthetic difference-in-differences counterfactual. In these plots, we show again that the gap is substantively small in 2020 and before.

### 3.3. Estimates of Effect of Grants on Democratic Vote Share and Turnout.

Next, we present our estimates of the effect of receiving a grant on turnout and Democratic vote share. Table 2 reports our estimates using a variety of estimation approaches. Column 1 is the simple difference-in-differences regression estimate of the effect of grant receipt on Democratic vote share. As we establish in Section 3.1, this is a dramatic overestimate of the effect because, even before 2020, Democrats were increasingly performing better in counties that received a grant than in counties that did not. In column 2, we present estimates from difference-in-differences regressions including time weights but not including county weights. The 2020 election result is much more similar to the 2016 result than any other previous period in our data, so the weight selection procedure places all of the mass on 2016 in the pretreatment period. This means that column 2 is equivalent to a two-group, two-period difference-in-differences design using only 2016 and 2020. With this estimator, we find that grants increase Democratic vote share by 0.36 percentage points. In column 3, we present our estimate from a difference-in-differences regression with county weights but no time weights. We find that, compared to counties that did not receive a grant but were on a similar average presidential voting trajectory prior to 2020, and after accounting for remaining pre-2020 average differences in Democratic vote share, grant recipients had only a 0.11-percentage-point higher Democratic vote share in 2020. Column 4 presents our difference-in-differences estimate using both time and county weights. Using this specification, we estimate that grants caused an average increase in Democratic vote share of 0.03 percentage points. In summary, once we compare grant recipients to more similar counties, we find that these grants did not substantially increase Democratic vote share in receiving counties.

**Table 2.** Election administration grants did not noticeably advantage democrats or increase turnout in 2020

	Dem vote share (%)				Turnout (%)			
	(1)	(2)	(3)	(4)	(5)	(6)	(7)	(8)
Grant recipient in 2020	3.26	0.36	0.11	0.03	0.24	0.14	−0.03	0.03
	(0.35)	(0.11)	(0.12)	(0.10)	(0.21)	(0.13)	(0.15)	(0.13)
Num grant recipients	924	924	924	924	924	924	924	924
Num counties	2,594	2,594	2,594	2,594	2,594	2,594	2,594	2,594
Observations	20,752	20,752	20,752	20,752	20,752	20,752	20,752	20,752
County FEs	Yes	Yes	Yes	Yes	Yes	Yes	Yes	Yes
Year FEs	Yes	Yes	Yes	Yes	Yes	Yes	Yes	Yes
County weights	No	No	Yes	Yes	No	No	Yes	Yes
Year weights	No	Yes	No	Yes	No	Yes	No	Yes

SE estimated with 1,000 county block bootstrap samples reported in parentheses. Data are a balanced panel of counties in the eight presidential elections from 1992 to 2020.

In columns 5 through 8, we present estimates of the effect of a grant on turnout using the same estimation strategies as in columns 1 through 4. Grant recipients and nonrecipients are on more similar turnout trajectories than Democratic vote trajectories prior to 2020. Accordingly, the four estimation strategies produce more similar results. In column 5, we present a likely upwardly biased 0.24-percentage-point difference-in-differences estimate of the effect of the grants on turnout. In columns 6 through 8 we find that, when we use time weights, county weights, or both, our estimates range from -0.03 percentage points to 0.14 percentage points.

Focusing on our preferred specification in columns 4 and 8 where we use both time and county weights in a difference-in-differences regression, we find that the grants did not substantially increase Democratic vote share or turnout. Our estimates are highly precise on both outcomes. The SE we report in column 4 is 0.10 percent, meaning that we could reject a hypothetical effect of the grants on Democratic vote share that is greater than 0.25 percentage points. Similarly, the SE we report in column 8 is 0.13 meaning that we could reject a hypothetical effect of 0.30 percentage points.

### 3.4. Estimated Effects Similar or Less Favorable to Democrats in Wisconsin.

In this section, we supplement our main finding with municipality-level election data from Wisconsin. We find that the grants did not substantially increase Democratic vote share or turnout.

As we discuss in Section 3.1, our main analyses study places where elections are administered at the county level because most parts of the country administer elections at that level, presidential election data are widely available at the county level, the population denominator is noisily measured in the many small municipalities, and changing municipal boundaries add potentially systematic noise to the election results data. Wisconsin is one of the states we leave out of this analysis because elections are primarily conducted at the municipal level (42). To validate our main findings, we constructed a separate dataset of municipal-level election results and grant receipt in Wisconsin. We provide more details on this dataset in *SI Appendix*, section S8.

Using the same estimation strategies we use in Table 2, we report estimates of the effect of private election administration grants in Wisconsin in *SI Appendix*, Table S5. We estimate very similar effects of the grants on Democratic vote share in Wisconsin as in our nationwide county-level analysis. Our preferred synthetic difference-in-differences estimator with county and year weights in column 4 estimates an effect of 0.11 percentage point. Across all three of our synthetic difference-in-differences estimates, our point estimates range from 0.11 to 0.31 percentage points, and we cannot reject the null hypothesis that the grants had no effect on Democratic vote share. The upper bound of the 95% CI on our preferred estimate is an effect of 0.48 percentage points.

In columns 6 through 8 of *SI Appendix*, Table S5, we report our estimates of the effect of grants on turnout in Wisconsin. Contrary to the expectation that grants increased turnout in Democratic strongholds and thereby advantaged Democrats, we find that municipalities that received grants had a modest but statistically significant drop in turnout of approximately 0.7 percentage points. We interpret this result as evidence against grants improving turnout in Democratic strongholds rather than strong evidence that the grants caused lower turnout in grant-receiving Wisconsin municipalities.

### 3.5. Estimated Effects Not Sensitive to Estimation Strategy.

We examine the robustness of our main estimates in Table 2 using five alternative estimation strategies. Across all five approaches, we find similar, substantively small effects of the grants on turnout and Democratic vote share. Our estimates of the average effect on Democratic vote share, reported in *SI Appendix*, Table S3 in the online appendix, range from 0.13 percentage points to 0.42 percentage points. Our estimates of the average effect on turnout, reported in *SI Appendix*, Table S4 also in the online appendix, range from −0.02 percentage points to 0.04 percentage points. These estimates come from five different strategies: 1) weighted regressions like synthetic difference-in-differences but without county-specific intercepts so the weights attempt to balance treatment and control outcomes on levels rather than trends, 2) regularized synthetic control (43), 3) weighting the control units such that pre-2020 control-group outcome means exactly match treatment means while deviating as little as possible from uniform weights (44), 4) predicting the outcome and treatment propensity using random forests and using these estimates for augmented inverse propensity weighting (45), and 5) predicting the outcome and treatment propensity using ensemble learners—pooling generalized additive models, boosting, regression trees, splines, and elastic nets—and using these predictions for augmented inverse propensity weighting.

As we discuss, in Section 2, many states passed laws in 2020 that may have affected how many of their counties applied for a grant and affected participation in the state. We can address this by comparing changes to Democratic vote share and participation within the same state. With our main estimators, it is infeasible to directly account for state-specific trends in Democratic vote share and turnout, but this is straightforward to do in a lagged-dependent-variable regression. In *SI Appendix*, Table S5, we present lagged-dependent-variable regression estimates of the average effect of a CTCL grant with and without state fixed effects. We document that including state fixed effects does not meaningfully change our estimates, implying that state-specific policy changes in 2020 are not substantially biasing our estimates of the effect of CTCL grants.

### 3.6. Estimated Effect Similar in Battleground States.

One concern is that, while the grants had a small effect on average, they may have had a larger effect in the closest states. We evaluate this claim by estimating the average effect of the grants on Democratic vote share and turnout in three sets of the most competitive states—states decided by less than 5 percentage points, states that the Cook Political Report identified as battleground states prior to election day, and Wisconsin alone.

In *SI Appendix*, Table S6 in the online appendix, we document that, even in the most competitive states, the effect of the grants was small. In column 1, we find that the effect of grants on Democratic vote share in close states was 0.54 percentage points. While this point estimate is larger than the effect we estimate using the full sample, the smaller sample size also means the subgroup estimates are substantially noisier, and we are unable to reject the hypothesis that the grants had no effect. When we extend our analysis to the battleground states according to the Cook Political Report, our estimate is more precise, and the estimated effect on Democratic vote share is nearly identical to our estimates from Table 2 using all counties.

In columns 3 and 4, we find that the effect of the grants on turnout in states decided by less than 5 percentage points and those the Cook Political Report labeled as battlegrounds was approximately the same size as we estimate using the full sample. In both cases, we cannot reject the hypothesis that the grants failed to increase turnout. We can also rule out positive effects on turnout of greater than 0.75 percentage points in the closest states.

Returning to the evidence we presented in Section 3.4, when we study the battleground state of Wisconsin independently, we find roughly the same pattern of small average effects, no average effects, or even negative average effects of the grants on Democratic vote share and turnout.

### 3.7. Estimated Effect Similar in Populous Counties.

Private election funding would have a larger effect on the aggregate election outcome if it was most effective in counties with more voters. Are the effects of grants larger in populous counties? We evaluate this possibility by splitting our sample into terciles by voting-age population and estimating effects for each subgroup separately. We present our results in *SI Appendix*, Table S7. We find that the effects on turnout and Democratic vote share are no larger in more and less populous counties.

### 3.8. Effects Not Larger for Counties Receiving Larger Grants.

If additional spending on local election administration increases turnout or Democratic vote share, this would most likely happen because local officials use the money to make it easier for citizens to participate. This implies that the effects of money should increase as they get larger or, at the very least, be unrelated to grant size. While CTCL reports using a formula to decide the maximum amount each county was eligible to receive, the amounts that CTCL distributed to counties ranged from $0.63 per voting-age resident at the 25th percentile to $1.38 per voting-age resident at the 75th percentile. Might our small effect estimates mask a more substantial effect in counties that receive larger grants?

To answer this question, we split treated counties into three groups based on the amount of grant money going to the county per voting-age resident and produce separate synthetic difference-in-differences estimates of the effect of small, medium, and large grants. We present the results of these analyses in *SI Appendix*, Table S8 in the online appendix. Contrary to the expectation that the effect may be limited to places receiving the largest grants, we find that small grants increased Democratic vote share and turnout by more than large grants. We estimate that small grants increased Democratic vote share by 0.63 percentage points and turnout by 0.31 percentage points while large grants decreased Democratic vote share by 0.59 percentage points and turnout by 0.26 percentage points. Given that, unlike our other analyses, the synthetic difference-in-differences weights do a poor job of matching pre-2020 treated and control trajectories for these subgroups, we also present estimates using entropy balancing to match pre-2020 outcome means for control counties to the treated county means (46). Using entropy balancing to balance on lagged outcomes, population, non-Hispanic White share, and non-Hispanic Black share, we no longer find negative effects for the largest recipients—the effects are almost exactly zero. We also find slightly smaller effects in places that received smaller grants. These effects average out to approximately the average effect estimates we present in Sections 3.3 and 3.5.

## 4. Characterizing the Magnitude of the Effects

How large are the effects of private election administration funding? In this section, we benchmark the magnitude of our effect estimates against the remarkably tight margin of the 2020 presidential election. The 2020 presidential election turned on four states decided by margins of 1.16 percentage points or less: Georgia, Arizona, Wisconsin, and Pennsylvania. The margins in these states were widely understood to be very tight. Are the effects of the private election administration grants as small or smaller than these margins?

One simple way to interpret the effect size is to compare the effect of private election funding on Democratic vote share to the margin in these four close states. Of our three main estimates reported in Table 2, two of them are too small to have changed the outcome in any state, including Georgia, Arizona, Wisconsin, and Pennsylvania. Our largest estimate is about as large as the margin in Wisconsin but still smaller than the margin in Pennsylvania. This is not to say that grant funding was sufficient to change the outcome in any of these states—only a subset of counties received the money, so county-level effects that are roughly the same magnitude as the margin in the state are not large enough to have swung the statewide outcome.

How large are the effects on turnout? One way to interpret these effects is to compare them to the effects of other election administration changes. The effect of the grants on turnout was less than half of the effect of an extra day of early voting (18), roughly half of the effect of a mailer encouraging citizens to vote by mail (47), less than one-tenth of the effect of universal vote by mail (16, 20), and less than one-twentieth of the effect of mobile voting (48). While the estimated effects of polling place locations on turnout are typically noised than our estimates of the effects of grants, our estimates of the effects of grants tend to be smaller than the effect of having your polling place moved further away (15, 19, 36). Our estimates of the effects of grants on turnout are small compared to all of these administrative changes.

Given the strong tendency of the grants to go to Democratic-leaning counties, the grants could advantage Democrats more than is implied by the Democratic vote share effect alone. On the other hand, many counties did not receive grants, so the effect of the grants on statewide totals is substantially smaller than the effect in the average county. To account for these concerns, we conducted a simple simulation study. In our simulation, we remove the average effect on turnout and Democratic vote share from all of the treated counties and assume untreated counties remain unchanged. (We also include municipal-level data from Wisconsin in our simulation analysis.) In states where we do not have grant data, we impute the probability that a county received a grant based on 2016 Democratic vote share and sample 1,000 random possible treatment assignments in those states. We then handle the counties we randomly assigned to treatment in that simulation as we handle the truly treated counties, removing the average effect of the grants on turnout and vote share.

Based on this simple simulation, we find that the estimates from two of our three weighted difference-in-differences estimation strategies imply that the grants were too small to swing the outcome of any statewide election. The estimates from our difference-in-differences strategy with time weights but not county weights are large enough to have changed the outcome of the election in Georgia and Arizona but inconsistent with changing the outcome of the election in Pennsylvania or Wisconsin. As we discuss above, this is our least plausible estimate because we have to assume that support for Democrats would have changed identically among grant-receiving and nonreceiving counties from 2016 to 2020. We take this as an upper bound and note that, even using our upper bound estimate, the effects we estimate are not large enough to have changed the outcome of the electoral college vote. Put together, this suggests that, even compared to the margin in very close elections recent elections, the effects of the grants were quite small.

It is important to note that, while these simulations help us understand the magnitude of the effects, we do not intend them as a reflection of what would have happened in the 2020 election had CTCL not made any grants. Our simulations do not account for the general equilibrium effects of the grants, such as changes in partisan spending, get-out-the-vote operations, or other government spending on election administration. Instead, we view our simulations as consistent with our interpretation that the grants had minimal effects.

## 5. Discussion

The large influx of private funding for election administration in 2020, and the fact that Democratic counties were more likely to receive it, has led many politicians, journalists, and pundits to speculate that the funding advantaged Democrats. Despite these widespread concerns, we present evidence that these grants did not substantially increase turnout or Democratic vote share. Our results answer one of the key questions at the center of the debate over private funding of election administration, suggesting that it does not always clearly and substantially favor one party.

Still, our findings leave unanswered two important questions: First, while we find that private funding did not increase turnout, it may have improved the election on other important dimensions. Many of the local officials who received the money said that they would have had trouble reporting their election results on time without the grants. Others said that the money allowed them to hire more staff which may have made the election run more smoothly, made voting more convenient, or improved election security and the accuracy of the count. We cannot observe these effects of the money, but they are important when deciding whether grant programs like these are effective. Second, the large backlash suggests that the grants may have led some citizens to doubt the outcome of the election. If that is the case, it is a potential cost worth considering in future attempts to shore up local election funding.

While our results suggest these grants did not substantially alter the outcome of the 2020 presidential elections, our results do not imply that a grant program like this cannot change election outcomes in the future. We understand our results to be only one input into a broader policy discussion about the appropriateness of grant programs like this given the potential positive effects grants could have on voter experience and security, the potentially harmful effects grants could have on citizen trust in elections, and the risk that future funders could alter election outcomes.

## 6. Materials and Methods

In Section 1.2, we discuss the data we collected to study the effect of CTCL grants on Democratic vote share and turnout. Our data on which counties received a CTCL grant comes from CTCL’s tax filings. Our election data come from Dave Leip’s Election Atlas. In Section 3.1, we discuss our main approach to estimating the effect of CTCL grants—synthetic difference-in-differences. We discuss the intuition for the approach as well as the formal details.

## Supplementary Material

Appendix 01 (PDF)

## Data Availability

County-level and municipal-level data on private election administration grant receipt, census data, and election outcomes all in tabular form have been deposited in Harvard Dataverse (https://doi.org/10.7910/DVN/BZW6AR) (49).

## Footnotes

^*^According to a 2022 Democracy Fund/Reed College survey of local election officials, 63% of local election officials “think that encouraging voter turnout is part of their job.” https://bipartisanpolicy.org/download/?file=/wp-content/uploads/2022/12/BPC_poster_Dec2022-FINAL-MMS.pdf.

^†^For example, one election official told the CTCL: “We are a small community struggling to find ways to handle unfunded mandates, especially during a pandemic. It means a lot to us to ensure our election process is done in all the right ways.” https://www.techandciviclife.org/grant-update-october/.

^‡^Beyond the CTCL funding, two other private grant programs ran in parallel in 2020: The USC Schwarzenegger Institute administered a much smaller (approximately $2.5 million) and narrowly targeted program, and the Center for Election Innovation and Research used donations from Zuckerberg and Chan to fund $50 million in total grants to state election officials. For a longer discussion, see ref. 28.

^§^Unless cited to a different source, we rely on the CTCL’s website for details about the grants (1).

^¶^The CARES Act sent approximately $400 million to states to fund election administration in 2020. These funds helped cover some of the unexpected costs associated with hold elections during the pandemic. According to a survey of local election officials, a majority of counties used CARES Act funds on measures to reduce COVID transmission at polling places, and many counties spent the funds to facilitate more mail voting (29).

^#^See this Brennan Center collection of arguments for more emergency funding in the 2020 election https://www.brennancenter.org/our-work/research-reports/election-officials-national-security-experts-and-business-leaders-support.

^‖^Alaska’s secretary of state reports election results at the election district level rather than the borough level, which is the equivalent of counties in Alaska and the level at which CTCL made grants in Alaska. We exclude Alaska from our data.

^**^These states are Connecticut, Maine, Massachusetts, Michigan, Minnesota, New Hampshire, Rhode Island, Virginia, Vermont, and Wisconsin.

^††^We exclude Cook, St. Clair, Vermilion, and Winnebago counties in Illinois and Jackson County in Missouri.

^‡‡^While not the primary objective of the paper and not identified by the research design, Suttmann-Lea and Merivaki (35) finds that CTCL grant recipients had higher absentee ballot rejection rates.

^§§^The FEC held that this fact was uncontested in MUR 7854. https://www.fec.gov/files/legal/murs/7854/7854_25.pdf., but every county and municipality did not apply for the funding. CTCL also asserts in FEC MUR 7584 that every eligible applicant received the amount they requested or more. They further state that many cases in which they gave a larger grant than requested were cases where jurisdictions asked for less than the $5,000 minimum grant. https://www.fec.gov/files/legal/murs/7854/7854_25.pdf.

^¶¶^See the case of Louisiana in which the Secretary of State encouraged applications while the Attorney General threatened to sue parishes that applied. https://www.theadvocate.com/batonrouge/news/politics/elections/mark-zuckerberg-funded-free-election-grants-draw-ire-of-jeff-landry-who-files-suit/articlee59425a0-08a2-11eb-9757-cba83bb12048.html.

^##^https://evic.reed.edu/wp-content/uploads/2023/11/EVIC_2023_LEO_Survey_Report.pdf.

^***^This approach, which they call synthetic difference-in-differences, builds on the synthetic control method and other related approaches (38–41).
