# Supplementary material for: Did private election administration funding advantage Democrats in 2020?
Source: Proc Natl Acad Sci U S A. 2024 May 21;121(22):e2317563121. doi: 10.1073/pnas.2317563121 (PMC11145248; doi:10.1073/pnas.2317563121)
Supplement: Supplementary file 1 — Appendix 01 (PDF) [file pnas.2317563121.sapp.pdf]

Online Appendix for Did Private Election Administration Funding Advantage Democrats in 2020?

Intended for online publication only.

Contents

S.1 New Laws Limiting Private Election Administration Grants 2

S.2 How Grant Funds Were Spent in Wisconsin 3

S.3 Decomposing Grant Selection 4

S.4 Variation in Share of Counties Receiving CTCL Grant across States 7

S.5 Validating Weighted Diff-in-Diff Using 2016 as a Placebo Treatment Period 8

S.6 Event Study Graph Documenting Minimal Effect of Grants on Democratic Vote Share and Turnout 9

S.7 Alternative Strategies for Estimating the Effect of Grants 10

S.8 Estimating the Effect of Grants in Wisconsin 12

S.9 Investigating How Grant Effects Vary Across Counties 14

S.9.1 Effect of Grants in Battleground States 14

S.9.2 Effect of Grants by County Population Tercile 15

S.9.3 Effect of Grants by Grant Size Tercile 16

S.1 New Laws Limiting Private Election Administration Grants

Figure S.1: **Twenty-Four States Have Passed Laws Limiting Private Election Administration Grants Since 2020.**

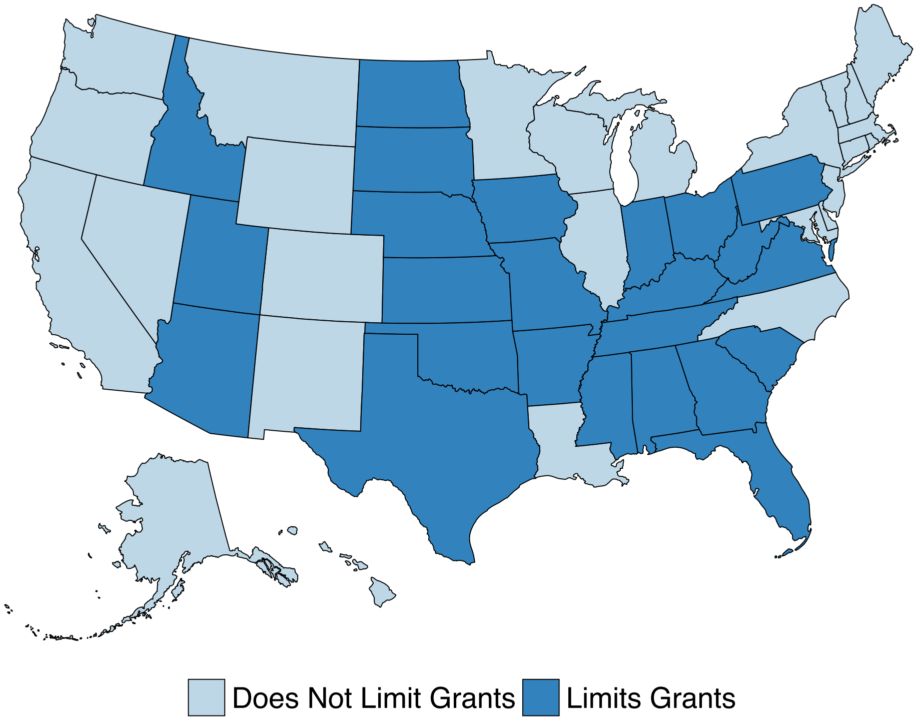

As we discuss in Section 1, twenty-four states have passed laws banning or substantially limiting private donations that support local election administration. Figure S.1 maps these states. Nearly every Southern state has passed a ban or limit on private funding. The only two Southern states that have not passed such a limit, Louisiana and North Carolina, have Democratic governors who vetoed legislative bills that introduced limits.

S.2 How Grant Funds Were Spent in Wisconsin

Figure S.2: **Wisconsin Municipalities Spent Majority of Funds on Staff and Equipment.**

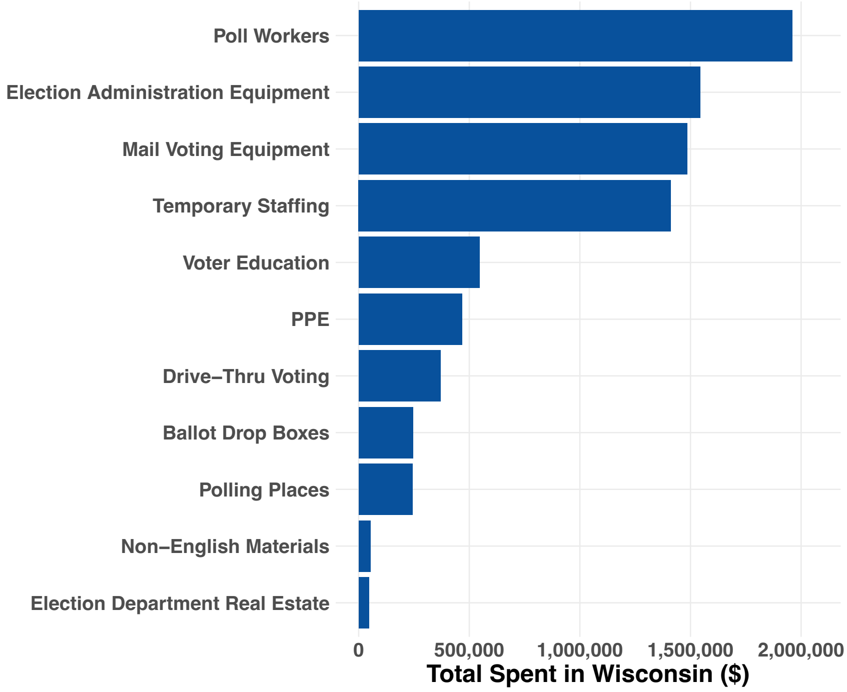

As we discuss in Section 2.1, we do not have comprehensive data on how the grant funds were spent. To get a sense for what spending categories local governments prioritized, we draw on data from the Wisconsin Institute for Law & Liberty. They used public records requests to obtain the local budgets describing how every CTCL grant recipient in Wisconsin planned to use their funds. We use their data to characterize how municipalities in Wisconsin spent their CTCL grant funds. Figure S.2 captures the spending totals by category. Wisconsin municipalities spent just over 40% of the funds on staff and just under 40% of the funds on equipment.

S.3 Decomposing Grant Selection

In Section 3 in the main text, we compare the types of counties that applied for funding to counties that did not apply. In this section, we explore the connection between mail balloting and applying for grant funding.

During the spring and summer of 2020, COVID killed many more people in Democratic-leaning counties than in Republican-leaning counties. Figure S.3 captures this pattern. Possibly for this reason as well as other psychological, sociological, political, and economic reasons, Democrats in the public were more eager to vote by mail in the 2020 election than were Republicans. Could this Democratic-Republican gap in demand for mail balloting in 2020 lead to different costs in Democratic and Republican counties and ultimately local officials to expect new costs and apply for a grant to cover them?

Figure S.3: **COVID Cases Primarily Concentrated in Clinton Counties Prior to 2020 Election.**

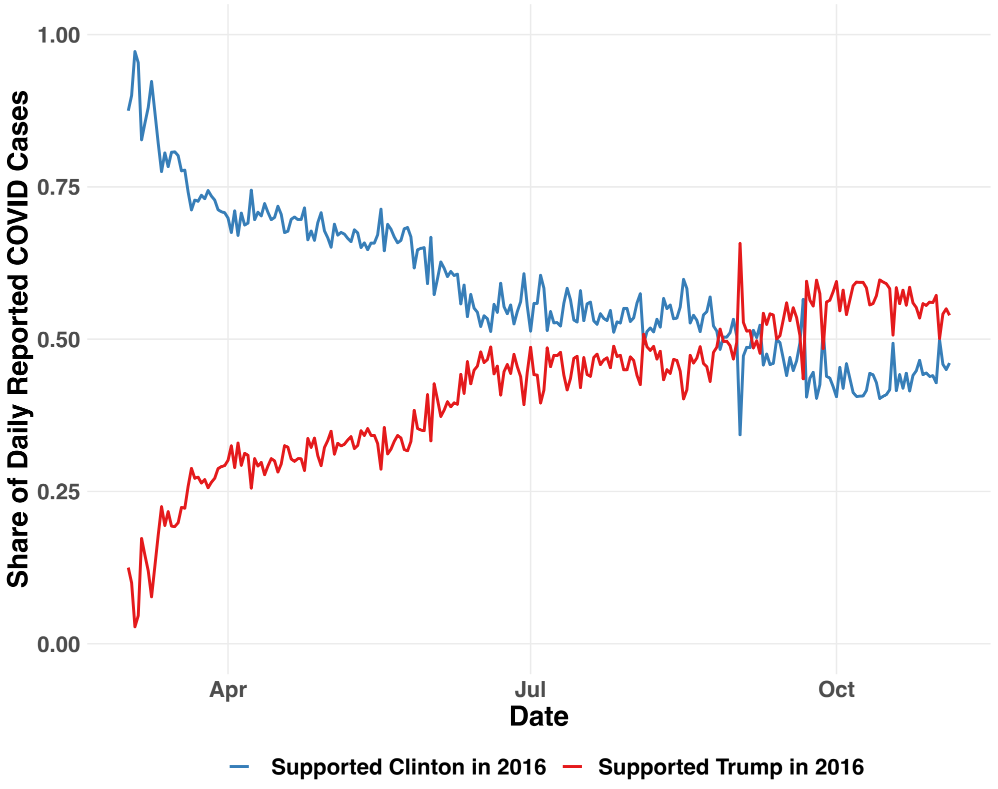

Table S.1: **In Democratic-Leaning Counties, More People Intended to Vote-by-Mail and Officials Were More Likely to Apply for CTCL Grant.**

	Mail Voting Share			Received Grant		
	(1)	(2)	(3)	(4)	(5)	(6)
Lag Dem Vote Share	0.23	0.33	0.09	0.43	0.15	0.44
	(0.04)	(0.06)	(0.07)	(0.11)	(0.13)	(0.15)
Lag Dem Vote Share × Mail Voting Expansion				0.54	0.35	0.26
				(0.18)	(0.18)	(0.17)
Mail Voting Expansion				-0.10	-0.01	
				(0.06)	(0.07)	
Observations	2356	2355	2355	1238	1237	1237
Controls	No	Yes	Yes	No	Yes	Yes
State FEs	No	No	Yes	No	No	Yes

Robust standard reported in parentheses. Mail voting share is the weighted share pre-election NationScape respondents in a county who said they intended to vote by mail. No-excuse expansion codes states that enacted no-excuse absentee voting for the first time in 2020 as 1. States that already had universal mail ballot delivery or offered absentee voting only with an excuse in 2020 are coded as 0. All other states are held out of this analysis. The controls are the log of voting age population, the log of median household income, and the non-Hispanic white share of the population.

In columns 1 through 3 of Table S.1, we present suggestive evidence that more citizens in Democratic-leaning counties were eager to vote by mail. We estimate the share of county registrants who intend to vote by mail using UCLA Nationscape survey questions about vote mode linked to the respondent's county. We find that counties that voted more Clinton in 2016 had substantially more residents saying they intended to vote by mail. This relationship shrinks considerably when we include state fixed effects that account different state election laws and is no longer statistically distinguishable from zero at conventional levels, but the point estimate is still positive.

In columns 4 through 6 of Table S.1, we present suggestive evidence that election officials in Democratic counties were more likely to apply for grants in part because they expected increased costs associated with expanding mail voting. We define three groups of states: those that only allow people to vote by mail with a special excuse, those that send every citizen a mail ballot, and those that removed the need for an excuse to vote by mail in 2020.

If officials in Democratic counties were more likely to apply for a grant in part because they anticipated increased mail voting costs, this relationship between Democratic vote share and applying for a grant should be larger in states that expanded mail voting than in states that maintained substantial restrictions on it or already mailed all registered voters a ballot. In column 4 we document that the relationship between Democratic vote share and grant receipt is substantially larger in states that expanded mail voting. In columns 5 and 6, we adjust for additional features of the counties and states. With these adjustments, the relationship between lagged Democratic vote share and grant receipt is more similar in states that expanded mail voting and those that did not, and the difference is no longer statistically distinguishable from zero at conventional levels, but we the point estimate is still positive and substantively large.

S.4 Variation in Share of Counties Receiving CTCL Grant across States

Figure S.4: **Share of Counties Receiving CTCL Grant by State.**

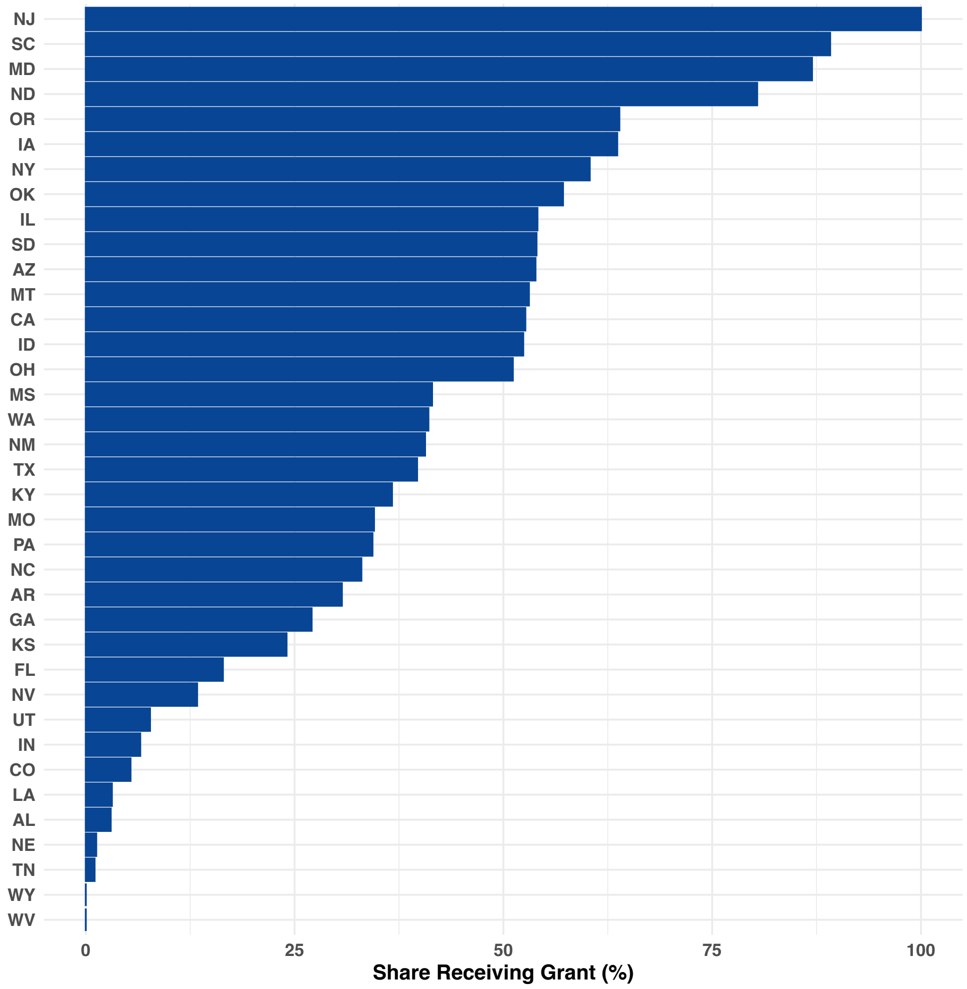

Figure S.4 presents the share of counties in each state that received a grant from CTCL. As we discuss in Section 3, while there is substantial variation across states in the share of counties that applied for a grant, Democratic-leaning counties are still substantially more likely to apply for a grant even when compared to Republican-leaning counties in the same state. One plausible explanation for this pattern is that Democratic-leaning counties expect more demand for COVID mitigation and use of mail voting than do Republican-leaning counties.

S.5 Validating Weighted Diff-in-Diff Using 2016 as a Placebo Treatment Period

Table S.2: **Weighted Difference-in-Differences Approach Balances Dem Vote Share and Turnout in 2016.**

	Dem Vote Share (%)				Turnout (%)			
	(1)	(2)	(3)	(4)	(5)	(6)	(7)	(8)
Grant Recipient in 2016	3.38	0.81	0.48	0.38	0.12	-0.14	-0.20	-0.17
	(0.38)	(0.21)	(0.22)	(0.22)	(0.17)	(0.10)	(0.10)	(0.10)
Num Grant Recipients	924	924	924	924	924	924	924	924
Num Counties	2,594	2,594	2,594	2,594	2,594	2,594	2,594	2,594
Observations	18,158	18,158	18,158	18,158	18,158	18,158	18,158	18,158
County FEs	Yes	Yes	Yes	Yes	Yes	Yes	Yes	Yes
Year FEs	Yes	Yes	Yes	Yes	Yes	Yes	Yes	Yes
County Weights	No	No	Yes	Yes	No	No	Yes	Yes
Year Weights	No	Yes	No	Yes	No	Yes	No	Yes

Standard errors estimated with 1,000 county block bootstrap samples reported in parentheses. Data is a balanced panel of counties in the 7 presidential elections from 1992 to 2016.

S.6 Event Study Graph Documenting Minimal Effect of Grants on Democratic Vote Share and Turnout

Figure S.5: **Gap in Democratic Vote Share and Turnout between Grant-Receiving and Synthetic Difference-in-Differences Counterfactual Over Time**

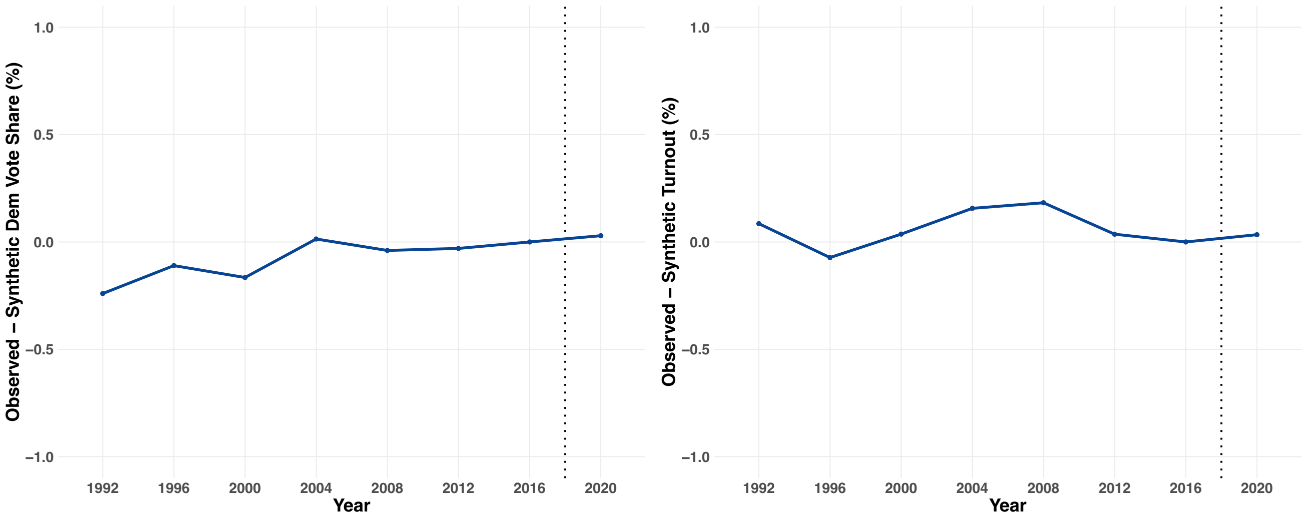

Figure S.5 plots the gap in Democratic vote share and turnout between the average grant recipient and the synthetic difference-in-differences counterfactual over time. This is the difference between the two lines in each plot in Figure 4. We can see that the change in Democratic vote share and turnout is small in 2020 relative to pre-treatment changes. We can also see that the synthetic difference-in-differences strategy produces a counterfactual that matches the grant recipient mean well in every pre-2020 period.

S.7 Alternative Strategies for Estimating the Effect of Grants

Table S.3: **Election Administration Grants Did Not Noticeably Advantage Democrats in 2020, Alternative Estimators.**

	Dem Vote Share (%)				
	(1)	(2)	(3)	(4)	(5)
Grant Recipient in 2020	0.30	0.42	0.25	0.27	0.13
	(0.13)	(0.77)	(0.11)	(0.11)	(0.08)
Num Grant Recipients	924	924	924	924	924
Num Counties	2,594	2,594	2,594	2,594	2,594
Observations	20,752	20,752	2,594	2,594	2,594
Estimator	SDID Without Intercept	Synthetic Control	Entropy Balancing	Causal Forest	Super Learner

Standard errors estimated with 1,000 county block bootstrap samples reported in parentheses. Data for columns 1, 2, 6, and 7 is a balanced panel of counties in the 8 presidential elections from 1992 to 2020. Data for columns 3, 4, 5, 8, 9, and 10 is wide with 7 lags of the dependent variable included. Synthetic control is a regularized synthetic control. SDID without intercept is synthetic difference-in-differences without county fixed effects. Entropy balancing is maximum entropy reweighting to balance grant recipients and non-recipients on the average of each lag of the outcome. Causal forest is double machine learning using random forests for both the outcome and propensity models. Super learner is double machine learning using an ensemble learner for both the outcome and propensity models.

Table S.4: **Election Administration Grants Did Not Noticeably Increase Turnout in 2020, Alternative Estimators.**

	Turnout (%)				
	(1)	(2)	(3)	(4)	(5)
Grant Recipient in 2020	0.03	-0.02	0.02	0.00	0.04
	(0.14)	(0.39)	(0.15)	(0.14)	(0.12)
Num Grant Recipients	924	924	924	924	924
Num Counties	2,594	2,594	2,594	2,594	2,594
Observations	20,752	20,752	2,594	2,594	2,594
Estimator	SDID Without Intercept	Synthetic Control	Entropy Balancing	Causal Forest	Super Learner

Standard errors estimated with 1,000 county block bootstrap samples reported in parentheses. Data for columns 1, 2, 6, and 7 is a balanced panel of counties in the 8 presidential elections from 1992 to 2020. Data for columns 3, 4, 5, 8, 9, and 10 is wide with 7 lags of the dependent variable included. Synthetic control is a regularized synthetic control. SDID without intercept is synthetic difference-in-differences without county fixed effects. Entropy balancing is maximum entropy reweighting to balance grant recipients and non-recipients on the average of each lag of the outcome. Causal forest is double machine learning using random forests for both the outcome and propensity models. Super learner is double machine learning using an ensemble learner for both the outcome and propensity models.

Table S.5: **Election Administration Grants Did Not Substantially Advantage Democrats or Increase Turnout in 2020, Within-State Estimates.**

	Dem Vote Share (%)		Turnout (%)	
	(1)	(2)	(3)	(4)
Grant Recipient in 2020	0.26	0.25	0.04	0.19
	(0.09)	(0.10)	(0.14)	(0.12)
Num Recipients	924	924	924	924
Num Counties	2594	2594	2594	2594
State FEs	No	Yes	No	Yes

Robust standard reported in parentheses. Each column reports estimates from a lagged dependent variable regression with lags from the seven prior presidential elections (1992 to 2016) included as regressors.

S.8 Estimating the Effect of Grants in Wisconsin

We supplement our main county-level analysis with a municipality-level analysis in Wisconsin. We built our main municipality-level analysis dataset from four sources: Wisconsin's Legislative Technology Services Bureau provided election results from 1990 to 2020 at the 2020 municipal ward level. We aggregate this data to the municipal level and link it to a list of all Wisconsin municipalities from Wisconsin's Department of Administration. We add grant amounts by hand to the list of municipalities with geocodes. Finally, we join this data with estimates of the voting age population in each municipality by mapping 2000 and 2010 Census block population statistics into 2020 Census blocks and aggregating to the municipal level. Given that we do not have visibility into how the Legislative Technology Services Bureau computed ward-level election results, we also replicate their work, collecting the original ward-level election results from 2004 to 2020. Our two election datasets are highly correlated and, in many cases, show the exact same number of votes for each party in the same municipality and year.

As we discuss in 4.4, Table S.6 captures our finding that the grants did not substantially increase Democratic vote share and, if anything, reduced turnout.

Table S.6: **Election Administration Grants Did Not Noticeably Advantage Democrats or Increase Turnout in Wisconsin in 2020.**

	Dem Vote Share (%)				Turnout (%)			
	(1)	(2)	(3)	(4)	(5)	(6)	(7)	(8)
Grant Recipient in 2020	1.33	0.31	0.24	0.11	-0.64	-0.71	-0.70	-0.69
	(0.58)	(0.20)	(0.19)	(0.19)	(0.40)	(0.28)	(0.28)	(0.29)
Num Grant Recipients	206	206	206	206	206	206	206	206
Num Counties	1,843	1,843	1,843	1,843	1,843	1,843	1,843	1,843
Observations	12,901	12,901	12,901	12,901	12,901	12,901	12,901	12,901
County FEs	Yes	Yes	Yes	Yes	Yes	Yes	Yes	Yes
Year FEs	Yes	Yes	Yes	Yes	Yes	Yes	Yes	Yes
County Weights	No	No	Yes	Yes	No	No	Yes	Yes
Year Weights	No	Yes	No	Yes	No	Yes	No	Yes

Standard errors estimated with 1,000 county block bootstrap samples reported in parentheses. Data is a balanced panel of counties in the 8 presidential elections from 1992 to 2020.

S.9 Investigating How Grant Effects Vary Across Counties

S.9.1 Effect of Grants in Battleground States

Table S.7: **Election Administration Grants Did Not Noticeably Advantage Democrats or Increase Turnout in 2020, Battleground States.**

	Dem Vote Share (%)		Turnout (%)	
	Close States	Cook Battlegrounds	Close States	Cook Battlegrounds
Grant Recipient in 2020	0.54	0.04	0.10	-0.46
	(0.29)	(0.20)	(0.31)	(0.22)
Num Grant Recipients	119	326	119	326
Num Counties	421	906	421	906
Observations	3,368	7,248	3,368	7,248
County FEs	Yes	Yes	Yes	Yes
Year FEs	Yes	Yes	Yes	Yes
County Weights	Yes	Yes	Yes	Yes
Year Weights	Yes	Yes	Yes	Yes

Standard errors estimated with 1,000 county block bootstrap samples reported in parentheses. Data is a balanced panel of counties in the 8 presidential elections from 1992 to 2020. Close states are those where the winner was decided by fewer than 5 percentage points. Cook battleground states are those that the Cook Political Report identified as battlegrounds prior to election day.

S.9.2 Effect of Grants by County Population Tercile

Table S.8: **Election Administration Grants Did Not Noticeably Advantage Democrats or Increase Turnout in 2020, Voting Age Population Tercile.**

	Dem Vote Share (%)			Turnout (%)		
	Small Population	Medium Population	Large Population	Small Population	Medium Population	Large Population
Grant Recipient in 2020	-0.24	0.15	-0.40	-0.71	0.08	-0.14
	(0.16)	(0.14)	(0.22)	(0.23)	(0.24)	(0.23)
Num Grant Recipients	256	253	415	256	253	415
Num Counties	865	865	864	865	865	864
Observations	6,920	6,920	6,912	6,920	6,920	6,912
County FEs	Yes	Yes	Yes	Yes	Yes	Yes
Year FEs	Yes	Yes	Yes	Yes	Yes	Yes
County Weights	Yes	Yes	Yes	Yes	Yes	Yes
Year Weights	Yes	Yes	Yes	Yes	Yes	Yes

Standard errors estimated with 1,000 county block bootstrap samples reported in parentheses. Data is a balanced panel of counties in the 8 presidential elections from 1992 to 2020. Small, medium, and large population counties are defined by terciles of voting age population.

S.9.3 Effect of Grants by Grant Size Tercile

In this section, we present estimates of the effect of CTCL grants by grant size tercile. Table S.9 presents our synth difference-in-differences estimates of both of our outcomes for each of the three treated groups. As we discuss in the body of the paper, synthetic difference-in-differences does not achieve sufficient balance because the penalized regression favors too much sparsity in the weights. We address this in Table S.10 by using entropy balancing weights in place of the unit weights selected by synthetic difference-in-differences. We also include the natural logarithm of the voting age population, the non-Hispanic white population share, and the non-Hispanic Black population share as additional variables on which to balance. In columns 1, 2, and 3, we find estimates of the effect of CTCL grants is similar regardless of grant size. In columns 4, 5, and, 6 we estimate that turnout declined in receiving counties by a very modest amount regardless of the size of the grant.

Table S.9: **Election Administration Grants Did Not Noticeably Advantage Democrats or Increase Turnout in 2020, Grant Size Tercile.**

	Dem Vote Share (%)			Turnout (%)		
	Small Grant	Medium Grant	Large Grant	Small Grant	Medium Grant	Large Grant
Grant Recipient in 2020	0.63	0.05	-0.59	0.31	0.06	-0.26
	(0.11)	(0.13)	(0.20)	(0.21)	(0.21)	(0.22)
Num Grant Recipients	308	308	308	308	308	308
Num Counties	1,978	1,978	1,978	1,978	1,978	1,978
Observations	15,824	15,824	15,824	15,824	15,824	15,824
County FEs	Yes	Yes	Yes	Yes	Yes	Yes
Year FEs	Yes	Yes	Yes	Yes	Yes	Yes
County Weights	Yes	Yes	Yes	Yes	Yes	Yes
Year Weights	Yes	Yes	Yes	Yes	Yes	Yes

Standard errors estimated with 1,000 county block bootstrap samples reported in parentheses. Data is a balanced panel of counties in the 8 presidential elections from 1992 to 2020. Grant sizes are determined by tercile of grant size per voting age resident among recipients. Small is the smallest tercile, and large is the largest tercile.

Table S.10: **Election Administration Grants Did Not Noticeably Advantage Democrats or Increase Turnout in 2020, Grant Size Tercile, Entropy Balancing Weights.**

	Dem Vote Share (%)			Turnout (%)		
	Small Grant	Medium Grant	Large Grant	Small Grant	Medium Grant	Large Grant
Grant Recipient in 2020	0.03	0.00	-0.23	-0.57	-0.54	-0.31
	(0.11)	(0.16)	(0.19)	(0.23)	(0.19)	(0.21)
Num Grant Recipients	308	308	308	308	308	308
Num Counties	1,978	1,978	1,978	1,978	1,978	1,978
Observations	13,846	13,846	13,846	13,846	13,846	13,846
County FEs	Yes	Yes	Yes	Yes	Yes	Yes
Year FEs	Yes	Yes	Yes	Yes	Yes	Yes
County Weights	Yes	Yes	Yes	Yes	Yes	Yes
Year Weights	Yes	Yes	Yes	Yes	Yes	Yes

Standard errors estimated with 1,000 county block bootstrap samples reported in parentheses. Data is a balanced panel of counties in the 8 presidential elections from 1992 to 2020. Grant sizes are determined by tercile of grant size per voting age resident among recipients. Small is the smallest tercile, and large is the largest tercile.
